# Semi-supervised medical image captioning via anatomical collaborative evidence network

**DOI:** 10.3389/fmed.2026.1816295

**Published:** 2026-05-26

**Authors:** Shengxiang Zhou, Qiurui Liu, Liping Cai, Ke Lu, Lushan Qiao, Nanbo Xu, Yunhan Wu, Yanhong Xu, Jun Li

**Affiliations:** 1 College of Information Engineering, Sichuan Agricultural University, Ya'an, China; 2 Department of Otolaryngology, Ya'an People's Hospital, Ya'an, China

**Keywords:** evidential deep learning, implicit anatomical localization, medical image captioning, Mixture-of-Experts (MoE), semi-supervised learning

## Abstract

**Introduction:**

Medical image captioning bridges visual perception and clinical language, but its development is limited by the high cost of detailed anatomical annotation and by the risk of hallucinations or overconfidence in ambiguous endoscopic images.

**Methods:**

We propose ACE-Net, an Anatomy Collaborative Evidence Network for semi-supervised medical image captioning. ACE-Net integrates evidential deep learning into the visual encoding stage through an evidence-driven soft-gating mechanism that quantifies epistemic uncertainty and suppresses unreliable visual noise. A triple-guided Mixture-of-Experts decoder further organizes clinical reasoning into semantic anchoring, visual evidencing, and spatial calibration. Spatial consistency alignment is imposed within a teacher-student co-training framework to promote stable anatomical attention patterns without pixel-level supervision.

**Results:**

On a high-resolution otolaryngology endoscopy dataset, ACE-Net achieved a BLEU-4 score of 0.7511 and a ROUGE-L score of 0.8728, demonstrating strong text-generation performance and improved anatomical grounding under limited annotation.

**Discussion:**

These results suggest that effective anatomical localization can be induced through evidence-constrained global supervision rather than expensive pixel-level annotations, providing a data-efficient and reliable paradigm for medical image captioning.

## 1 Introduction

Medical image captioning, as a translation between visual data and clinical linguistics, aims to automate accurate diagnostic descriptions, thereby reducing the workload of relevant physicians. The field is currently witnessing a paradigm shift due to the explosive capabilities of large-scale visual-language pre-training (VLP) frameworks such as CLIP (1), BLIP (2), and BLIP-2 (3). The release of numerous biomedical archives, such as BIOMEDICA (4), has further fueled ambitions to apply these multimodal foundational models to the medical field. However, a key gap remains: while foundational models perform well in generalization, they often underperform when dealing with the delicate, nuanced anatomical foundations required in specialized areas such as gastrointestinal or ENT endoscopy. The current challenge lies primarily in the inherent differences in the data images themselves. Unlike radiological images (e.g., X-rays, MRIs), which typically have stable illumination and structural consistency, endoscopic images are inherently cluttered. As Ali et al. (5) have noted, these images are frequently affected by severe artifacts, including specular reflections, fluid obstruction, and motion blur. While recent Transformer-based attempts (6, 7) aim to achieve automatic caption generation in this field, relying solely on deterministic feature extraction in such noisy environments carries significant risks. Standard models, lacking mechanisms for handling uncertainty, may misinterpret visual imperfections (such as blood pools) as pathological lesions, leading to dangerous “hallucinations” and unnecessary overconfidence.

**Figure 1:**
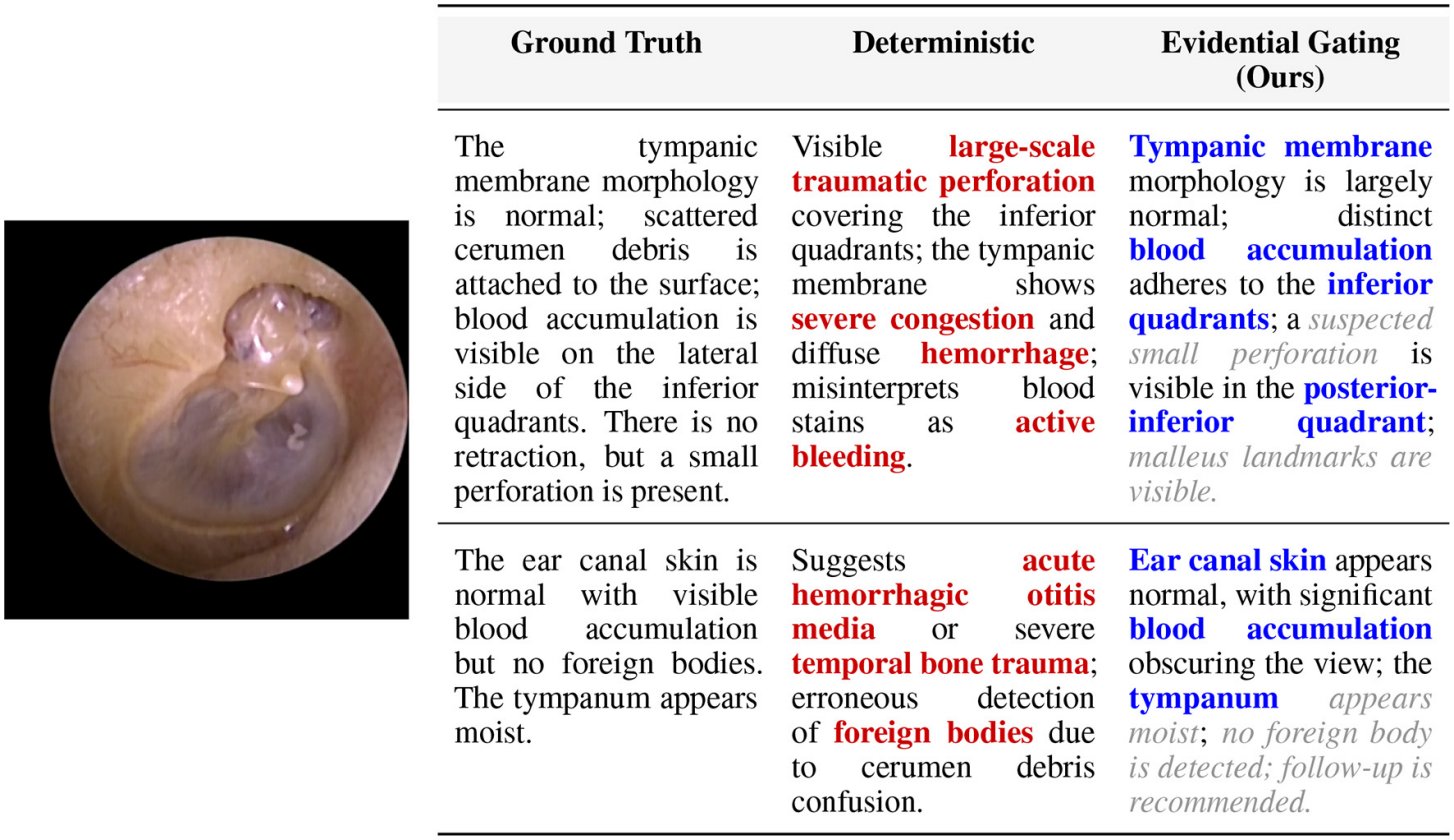
An illustrative example showing evidence-driven gating helps mitigate diagnostic hallucinations. Deterministic models suffer from “over-confidence,” often misinterpreting artifacts (e.g., blood accumulation) as severe lesions. In contrast, the evidential gating mechanism effectively filters unreliable features, generating more cautious and accurate reports.

As illustrated in Figure 1, are presentative otoscopic case demonstrates how this problem arises in practice. Compared with the ground-truth description, the deterministic model over-interprets visually ambiguous findings, such as blood accumulation and cerumen debris, as severe traumatic perforation, active bleeding, or foreign bodies. In contrast, the evidential gating strategy produces a more cautious and visually grounded description by preserving reliable anatomical evidence while suppressing uncertain artifact-related features. This example motivates the use of spatial uncertainty modeling as a confidence filter in ACE-Net.

To mitigate these risks, integrating uncertainty estimation has become a promising frontier. Representative works like Zheng He's “Evidence-Guided Learning” (EIL) (8) successfully leverage evidence theory to quantify uncertainty in interactive caption generation. However, EIL primarily treats uncertainty as a scalar indicator of keyword selection, ignoring its spatial distribution. Consequently, it lacks an intrinsic mechanism for suppressing local visual noise during the encoding phase, making the model susceptible to overconfidence traps in blurred anatomical regions. To address these limitations, we propose the “Anatomy Collaborative Evidence Network” (ACE-Net), a semi-supervised framework designed for reliable report generation and implicit anatomical localization without relying on pixel-level masks. Our approach advances the field through two architectural innovations:

First, we extend Evidence-Driven Deep Learning (EDL) to the spatial domain via an “evidence-driven soft-gating” mechanism. By computing pixel-level “hole” maps, this module dynamically reduces feature weights in high-uncertainty regions, effectively filtering out artifacts (5).

Second, we introduce a “triple-guided expert mixer” (MoE) decoder. Unlike standard causal decoders, this module coordinates a hierarchical reasoning flow—from predicted topic to global visual context, and finally to Local Anatomical Attention Map (AAM)—mimicking a structured clinical reasoning process (9).

Crucially, ACE-Net implements implicit anatomical learning. Instead of relying on expensive bounding box annotations, our model employs teacher-student consistency constraints, ensuring that stable anatomical heatmaps emerge only under explanatory text-level supervision.

Our main contributions are summarized below:

- We propose a unified framework, **ACE-Net**, that synergistically combines evidence uncertainty modeling and semi-supervised learning to effectively reduce hallucinations in endoscopic report generation.
- This paper designs a novel **evidence-driven soft-gating** module that explicitly suppresses spatial visual noise (such as specular reflections) by quantizing feature-level vacuum.
- We developed a **triple-guided MoE decoder** that sequentially integrates topic intent, visual evidence, and anatomical signals, significantly enhancing the logical consistency of the report.
- Experimental results show that ACE-Net demonstrates advantages in both annotation accuracy and data efficiency compared to existing methods.

### 1.1 Related work

#### 1.1.1 Medical report generation and vision-language pre-training

The domain of medical report generation has evolved rapidly, progressing from early template-based systems to deep learning architectures (9, 10). In parallel with the widespread adoption of deep convolutional networks for high-accuracy computer-aided diagnosis in medical imaging (11), initial approaches primarily adapted CNN-RNN frameworks from natural image captioning (12, 13). However, these methods often struggled with the long-tail distribution of medical terms. To address this, recent studies have pivoted toward Transformer-based architectures (14, 15), introducing diverse attention mechanisms—such as cross-modal feature fusion (16), hierarchical task structures (17), and memory-driven retrieval (7)—to enhance semantic alignment. Furthermore, multi-scale encoder-decoder self-attention mechanisms have demonstrated the significant advantage of explicitly capturing global and local contexts simultaneously across different representational abstractions (18). Specialized techniques like contrastive learning (19, 20) and hard-negative mining (21) have further improved the discriminative power of these models on rare pathological cases.

Concurrently, the field has been reshaped by large-scale Vision-Language Pre-training (VLP). Foundation models like CLIP (1), BLIP (2), and BLIP-2 (3) have demonstrated remarkable zero-shot transfer capabilities. In the biomedical sphere, initiatives like BIOMEDICA (4) have scaled this paradigm to millions of image-text pairs. Despite these advancements, standard VLMs often lack robustness when facing domain-specific visual noise (e.g., endoscopic artifacts) (5). Moreover, purely retrieval-based or generative models without explicit constraints are prone to “hallucinating” non-existent lesions (22, 23). Unlike these approaches, ACE-Net specifically targets the suppression of such visual noise through an evidence-based mechanism.

#### 1.1.2 Uncertainty quantification and evidential deep learning

Reliability and trustworthiness are non-negotiable in clinical AI. While standard deep networks provide point estimates, they often exhibit unwarranted over-confidence on Out-of-Distribution (OOD) data (24). Uncertainty Quantification (UQ) aims to mitigate this risk. Although ensemble methods and Monte Carlo Dropout offer solutions, they incur high computational overhead (25). Consequently, Evidential Deep Learning (EDL), grounded in Subjective Logic, has gained traction for its ability to estimate epistemic uncertainty in a single forward pass (26). In medical imaging, recent works have begun to integrate EDL for reliable classification and domain adaptation (27, 28). For captioning tasks, Zheng and Yu (8) proposed Evidence-Informed Learning (EIL) to guide human-in-the-loop interactions. Wang et al. (29) also explored semantic consistency to quantify uncertainty for hallucination detection. However, most existing methods treat uncertainty as a sample-level metric for abstention or weighting. They rarely exploit the *spatial distribution* of uncertainty (vacuity) to explicitly filter local visual artifacts during the encoding phase. ACE-Net bridges this gap by employing a pixel-wise vacuity map as a soft-gating mechanism, effectively “denoising” the visual representation before it reaches the decoder.

#### 1.1.3 Mixture-of-Experts in medical reasoning

To handle the heterogeneity of medical data, Mixture-of-Experts (MoE) architectures have become a prominent research direction. By decomposing complex tasks into specialized sub-networks, MoE models can adaptively process diverse inputs. For instance, MedMoE (30) and METransformer (31) utilize expert tokens to manage different imaging modalities or learn complementary feature subspaces. More recently, Peltekian et al. (32) introduced Regional Expert Networks (REN), which assign experts to specific anatomical regions (e.g., lung lobes) based on hard priors. While these methods improve feature diversity, they often overlook the *sequential nature* of the diagnostic workflow. clinicians typically reason in stages: identifying semantic topics, verifying visual details, and confirming spatial locations (33). Existing MoE routers generally operate in parallel or are conditioned solely on data modality. In contrast, our Triple-Guided MoE simulates the clinical reasoning chain. By cascading semantic anchors, visual evidence, and spatial calibration, our decoder ensures that the generated report is logically coherent and anatomically grounded.

## 2 Materials and methods

### 2.1 Problem formulation

We frame medical report generation as a semi-supervised sequence learning task, specifically designed to extract generalized anatomical representations from a landscape where raw data is abundant but expert annotation is scarce. Formally, let I denote the image space and Y the target report space. The dataset D is divided into two distinct subsets:

- A labeled set $D_{L}={\left\{\left(I_{i},Y_{i}\right)\right\}}_{i=1}^{N_{L}}$, where each image *I*_*i*_ is paired with a diagnostic report *Y*_*i*_. Crucially, we operate under a **weakly supervised localization setting**, meaning no pixel-level annotations (e.g., masks or bounding boxes) are provided.
- An unlabeled set $D_{U}={\left\{I_{j}\right\}}_{j=1}^{N_{U}}$, consisting solely of raw endoscopic images without any textual descriptions.

Our objective is to learn a mapping function $f_{\theta }:I\to Y$ that maximizes the likelihood of generated sequences on $D_{L}$, while simultaneously inducing implicit anatomical alignment through consistency constraints on the unlabeled distribution $P\left(D_{U}\right)$.

### 2.2 The ACE-Net architecture

**Figure 2:**
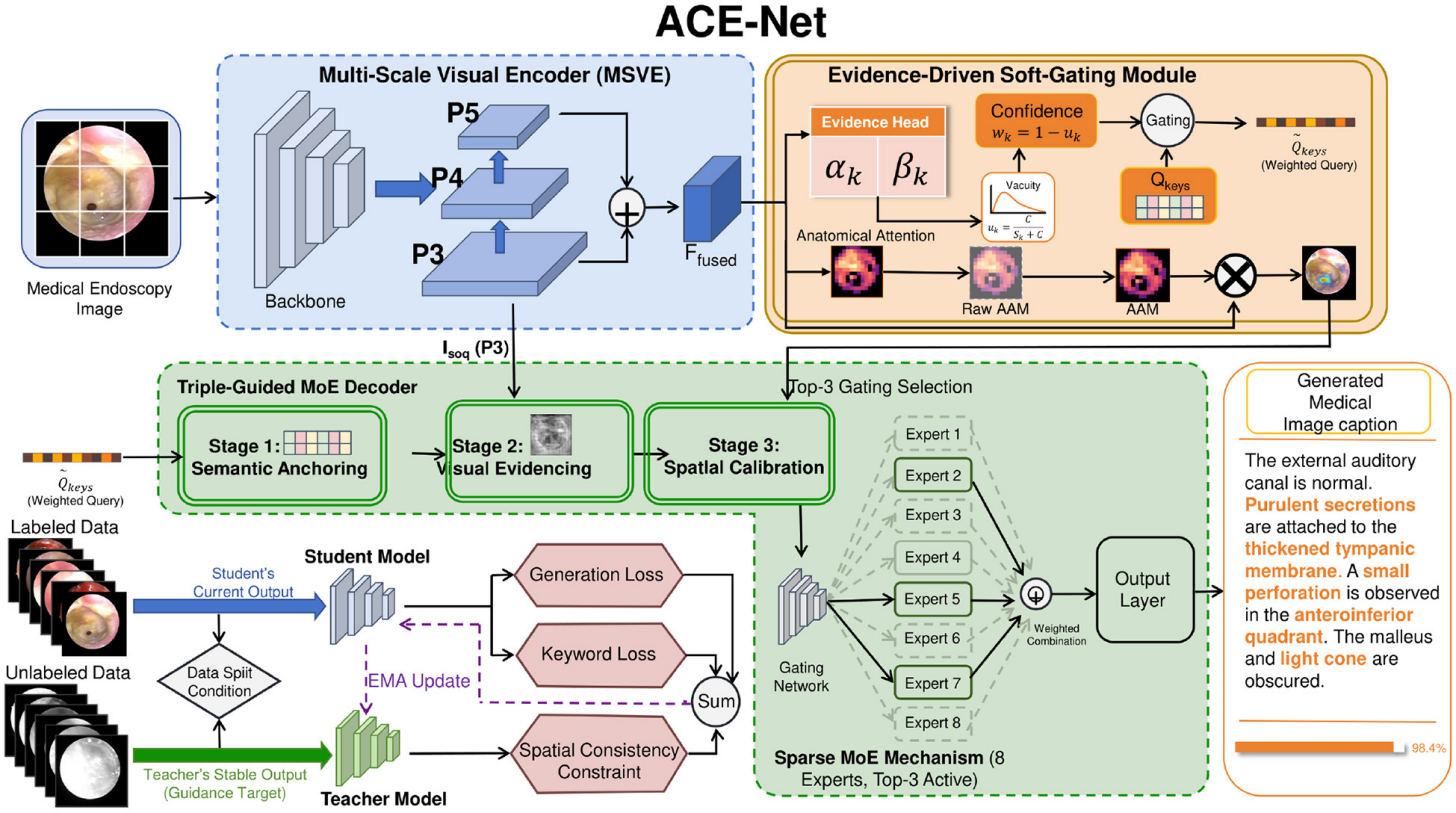
Overview of the ACE-Net architecture. The framework integrates three collaborative components within a teacher-student semi-supervised paradigm: (1) A **Multi-Scale Visual Encoder** fuses pyramidal features (*P*3-*P*5) to generate a global visual representation *F*_*fused*_. (2) An **Evidence-Driven Soft-Gating Module** regulates feature flow by quantifying epistemic uncertainty (*u*_*k*_). It explicitly constructs **Weighted Query Vectors (**${\tilde{Q}}_{keys}$**)** and **Filtered Features** (calculated via the element-wise product of *F*_*fused*_ and AAM) to suppress visual noise. (3) A **Triple-Guided MoE Decoder** hierarchically utilizes these signals across three stages: *Semantic Anchoring* (using ${\tilde{Q}}_{keys}$), *Visual Evidencing* (using Filtered Features), and *Spatial Calibration* (using AAMs). Additionally, a spatial consistency constraint is imposed between the teacher and student networks to effectively leverage unlabeled data.

As illustrated in Figure 2, ACE-Net transcends the conventional encoder-decoder paradigm by embedding uncertainty awareness directly into the clinical reasoning workflow. The architecture is orchestrated across three collaborative phases, mirroring the cognitive process of a radiologist:

1. **Perception phase: multi-scale visual encoder (MSVE)**. To capture lesions that vary drastically in scale within endoscopic views, we employ a Feature Pyramid Network (FPN) backbone (ResNet-101). This module fuses deep semantic features (*P*_5_) with shallow textural details (*P*_3_). This results in a comprehensive visual representation *F*_*fused*_ that simultaneously captures global anatomical contours and fine-grained pathological textures.
2. **Discrimination phase: evidence-driven soft-gating**. Situated as an adaptive bottleneck between the encoder and decoder, this module leverages Evidential Deep Learning (EDL) to quantify the “vacuity” (epistemic uncertainty) of local features. By generating a spatial confidence map, it dynamically re-weights *F*_*fused*_, effectively suppressing visual noise—such as specular reflections—in high-uncertainty regions while preserving salient diagnostic signals.
3. **Expression phase: triple-guided MoE decoder**. We replace the standard causal decoder with a sparse Mixture-of-Experts (MoE) architecture. This design simulates a hierarchical reporting process driven by three distinct guidance signals: (i) **Semantic anchors** (*Q*_*keys*_) for thematic intent; (ii) **Visual evidence** (*I*_*seq*_) for texture verification; and (iii) **Spatial calibration** (*A*_*map*_) for anatomical grounding.

### 2.3 Uncertainty modeling via evidence theory

Standard deep learning classifiers typically rely on Softmax functions, which yield point estimates of class probabilities. However, this approach inherently limits the model's ability to distinguish between “low evidence” (ignorance) and “conflicting evidence” (ambiguity), often leading to over-confident predictions on noisy endoscopic images. To address this, we incorporate **Evidential Deep Learning (EDL)** to explicitly quantify epistemic uncertainty.

#### Evidence formulation

**Figure 3:**
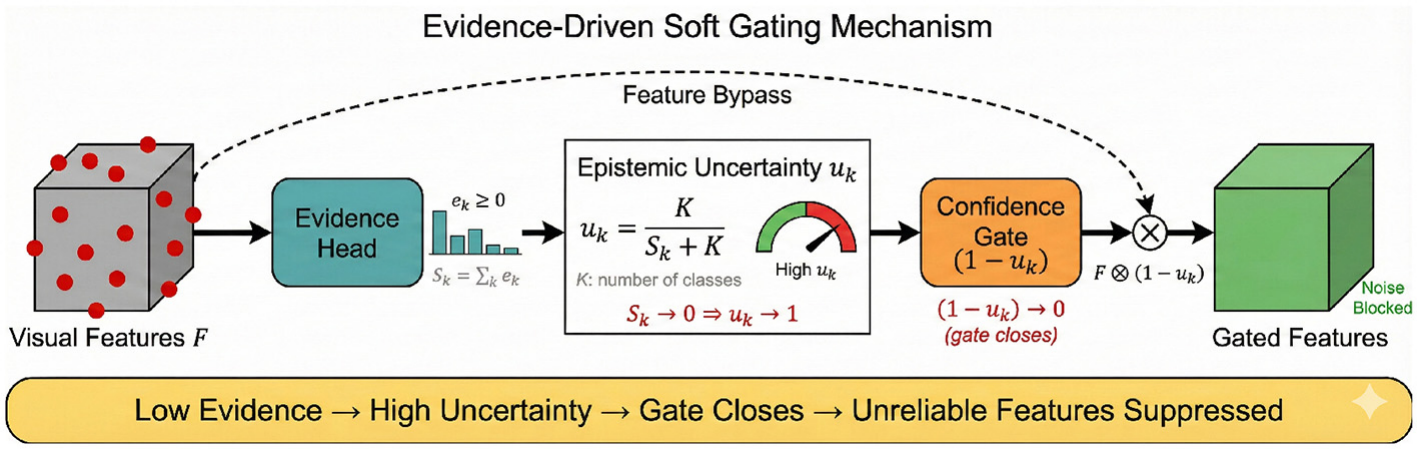
Schematic of the Evidence-Driven Soft Gating mechanism. We model the presence of each anatomical attribute as a Beta distribution. The module predicts evidence parameters (α, β) to compute the vacuity score *u*. This score acts as a differentiable confidence gate, suppressing features associated with high epistemic uncertainty.

As shown in Figure 3, we reformulate the detection of each anatomical keyword as a binary evidence accumulation process. For the *k*-th keyword (e.g., “Perforation”), the network predicts a pair of evidence parameters ${\text{e}}_{k}=\langle e_{k}^{pos},e_{k}^{neg}\rangle \geq 0$, representing the support for its presence and absence, respectively. According to Subjective Logic (SL), these evidence values are mapped to the parameters of a **Beta distribution** *Beta*(α_*k*_, β_*k*_), where $\alpha_{k}=e_{k}^{pos}+1$ and $\beta_{k}=e_{k}^{neg}+1$. The total evidence strength is defined as *S*_*k*_ = α_*k*_+β_*k*_. While the theoretical epistemic uncertainty (or “vacuity”) is typically defined as 2/*S*_*k*_, such a formulation can be numerically unstable in high-dimensional multi-label settings. To mitigate this, we introduce a scaling factor *C* (where *C* equals the total number of keywords *K*) to act as a smoothing prior. The optimized uncertainty *u*_*k*_ is derived as:


$$\begin{array}{l} u_{k}=\frac{C}{S_{k}+C} \end{array}$$ (S1)

This formulation effectively stabilizes gradients when evidence is extremely sparse, ensuring robust convergence during the early stages of training.

#### Evidential soft-gating

The derived uncertainty metric *u*_*k*_∈[0, 1] serves as a proxy for the reliability of visual features associated with the *k*-th anatomical concept. When an image region contains severe artifacts (e.g., specular reflections) that obscure the anatomical structure, the network struggles to collect valid evidence, causing *S*_*k*_ to remain small and *u*_*k*_ → 1. To exploit this signal, we design a differentiable gating mechanism. We first define a confidence score *w*_*k*_ = 1−*u*_*k*_. This score is then used to modulate the specific anatomical query embeddings *Q*_*keys*_:


$$\begin{array}{l} {\tilde{Q}}_{keys}^{\left(k\right)}=w_{k}\cdot Q_{keys}^{\left(k\right)} \end{array}$$ (S2)

In addition to modulating queries, we derive **Filtered Features** *F*_*filtered*_ to further refine the visual representation. This is achieved via the element-wise product of the fused visual features *F*_*fused*_ and the Anatomical Attention Maps (AAM):


$$\begin{array}{l} F_{filtered}=F_{fused}⊙\text{AAM} \end{array}$$ (S3)

Physically, this dual operation functions as a **confidence filter**: it actively suppresses retrieval queries and filters out visual tokens for anatomical regions deemed “unknowable” (i.e., high vacuity). This prevents the decoder from generating **hallucinations** (previously referred to as “illusions”) based on visual noise and ensures that the model focuses only on regions with sufficient evidential support.

### 2.4 Triple-Guided Mixture-of-Experts decoder

To emulate the hierarchical workflow of clinicians—who typically formulate a hypothesis, verify visual details, and then confirm anatomical locations—we redesign the decoding stage. Standard Transformer decoders rely on a single cross-attention mechanism, which often leads to modality entanglement. In contrast, we propose a **Triple-Guided Mixture-of-Experts (MoE)** architecture that decouples clinical reasoning into three sequential stages.

#### Cascaded reasoning flow

Structurally, each decoder layer incorporates three specialized Multi-Head Cross-Attention (MHCA) modules arranged in a cascade. Formally, let $H_{0}^{\left(l\right)}$ be the self-attention output of the *l*-th layer. The reasoning state is updated sequentially via:


$$\begin{array}{l} H_{t}^{\left(l\right)}=\text{MHCA}\left(H_{t-1}^{\left(l\right)},C_{t}\right),\;\text{for}t\in \left\{1,2,3\right\} \end{array}$$ (S4)

where *C*_*t*_ represents the distinct context guidance for each stage:

1. **Stage 1: Semantic anchoring (*C*_1_ = *Q*_*keys*_)**. The decoder first queries the *Key Topics* generated by the evidence module. This step establishes the high-level diagnostic intent and filters out irrelevant concepts based on confidence scores.
2. **Stage 2: Visual evidencing (*C*_2_ = *I*_*seq*_)**. Conditioned on the semantic context, the model attends to the dense visual feature sequence (*P*_3_ features). This step retrieves specific textural details (e.g., “congestion”) to substantiate the hypothesis.
3. **Stage 3: Spatial calibration (*C*_3_ = *A*_*map*_)**. Finally, the decoder attends to the Anatomical Attention Maps. This unique step forces the model to verify *where* it is looking, explicitly aligning the generated text with implicit anatomical heatmaps to prevent spatial hallucinations.

#### Sparse MoE with load balancing

To handle the high variability of medical terminology, we replace the standard Feed-Forward Network (FFN) with a sparsely gated Mixture-of-Experts layer. Let ${\left\{E_{i}\right\}}_{i=1}^{N}$ denote a set of *N* expert networks. For an input token representation *x*, a gating network *G*(·) activates the Top-*k* experts:


$$\begin{array}{l} y=\underset{i\in K}{\sum }w_{i}\cdot E_{i}\left(x\right),\;\text{where}K=\text{Top-}k\left(G\left(x\right)\right) \end{array}$$ (S5)

Here, *w*_*i*_ is the gating weight normalized via Softmax. In our implementation, we set *N* = 8 and *k* = 3. To prevent “expert collapse”—where the gate converges to selecting only a few experts—we introduce an auxiliary **Load Balancing Loss** ($L_{aux}$). This constraint encourages a uniform distribution of expert utilization across the batch, ensuring the model retains diverse descriptive capabilities for long-tail pathological cases.

### 2.5 Co-training via spatial consistency alignment

To circumvent the dependency on large-scale annotated data, we establish a semi-supervised framework based on the Mean Teacher paradigm. Unlike traditional methods that rely on noisy pseudo-captions, we propose a **Spatial Consistency Alignment** strategy. We postulate that while generating perfect text for unseen images is difficult, the underlying *anatomical attention patterns* should remain stable between the student and teacher models.

#### Dual-path optimization

The training process minimizes a joint objective derived from two parallel pathways:

**1. Supervised path (labeled data)**. On the labeled set $D_{L}$, the Student model (*S*) minimizes a composite supervised loss $L_{sup}=L_{gen}+\lambda_{key}L_{key}$, where $L_{gen}$ and $L_{key}$ denote the cross-entropy losses for caption generation and anatomical keyword classification, respectively.

**2. Consistency path (unlabeled data)**. On the unlabeled set $D_{U}$, we enforce the Student to align its **Anatomical Attention Maps (AAMs)** with those of the Teacher model (*T*). To prevent the propagation of noise, we weight this consistency by the Teacher's confidence. The spatial consistency loss is defined as:


$$\begin{array}{l} L_{ssl}={𝔼}_{x\sim D_{U}}\left[||\left(1-u_{T}\right)\cdot \left(A^{S}\left(x\right)-A^{T}\left(x\right)\right){||}_{2}^{2}\right] \end{array}$$ (S6)

where *u*_*T*_ is the Teacher's epistemic uncertainty. This mechanism ensures that consistency is enforced strictly only when the Teacher is confident about the anatomical structure.

#### Total objective

The overall framework is optimized end-to-end by minimizing the total loss $L_{total}$:


$$\begin{array}{l} L_{total}=\underset{\text{Supervised}}{\underset{︸}{L_{gen}+{\text{λ}}_{key}L_{key}}}+{\text{λ}}_{ssl}\underset{\text{Unsupervised}}{\underset{︸}{L_{ssl}}}+{\text{λ}}_{aux}L_{aux} \end{array}$$ (S7)

During training, the Student parameters are updated via gradient descent, while the Teacher parameters are updated as an Exponential Moving Average (EMA) of the Student to ensure temporal stability.

### 2.6 Dataset and implementation details

#### Dataset acquisition and protocol

The clinical endoscopy dataset utilized in this study was exclusively collected from the Department of Otolaryngology at **Ya'an People's Hospital**. The corpus reflects a realistic clinical distribution, comprising a total of 7,328 high-resolution otolaryngologic endoscopy images. Following a semi-supervised protocol, we partitioned the data into a labeled set of *N*_*L*_ = 1, 011 image-text pairs and an abundant unlabeled set of *N*_*U*_ = 6, 317 images. All raw images were resized to 224 × 224 pixels and normalized. To ensure a rigorous evaluation, we employed stratified sampling to reserve 102 samples from $D_{L}$ as an independent test set, ensuring that the distribution of pathological categories (e.g., Perforation, Otitis Media) remains consistent with the training distribution.

#### Public benchmark dataset: IU-Xray

To explicitly address the generalizability and data efficiency of our model across different medical domains, we additionally evaluated ACE-Net on the widely adopted Indiana University Chest X-Ray (IU-Xray) dataset. To rigorously simulate a data-scarce clinical environment, we partitioned the entire dataset into two equal halves: 50% as the unlabeled set and 50% as the labeled set. The labeled set was further divided into training, testing, and validation sets with a ratio of 7:2:1, respectively. Consequently, our semi-supervised ACE-Net was optimized using only 35% (i.e., 70% of the 50% labeled subset) of the total dataset for text-level supervision, relying on spatial consistency constraints to leverage the remaining unlabeled data.

#### Implementation setup

All experiments were implemented in PyTorch on a single NVIDIA GPU. We leveraged an ImageNet-pretrained ResNet-101 as the visual backbone, projecting features into a 512-dimensional space to capture robust initial representations. The MoE decoder was instantiated with 8 experts, utilizing a Top-3 sparse activation mechanism to ensure sufficient representational diversity without incurring excessive computational cost. Optimization relied on the Adam algorithm with an initial learning rate of 5 × 10^−5^, dynamically adjusted via a ReduceLROnPlateau scheduler to fine-tune convergence. To maintain stability during the semi-supervised co-training phase, the teacher model evolved via Exponential Moving Average (EMA) with a decay factor of α = 0.999. To ensure a fair and rigorous evaluation of cross-domain generalizability, consistent hyperparameter settings were applied across both the otolaryngology and IU-Xray datasets. Specifically, the models were trained for 100 epochs with a batch size of 32. Crucially, we adopted a “semantic-first” weighting strategy (λ_*Key*_≈10.0), prioritizing the accurate alignment of anatomical keywords early in training to anchor the subsequent caption generation effectively.

## 3 Results

### 3.1 Main results: comparative performance analysis

We benchmarked ACE-Net against a spectrum of established baselines, ranging from early CNN-RNN architectures (SAT, R2GenCNN) to recent Transformer-based (R2Gen) and multi-task paradigms (M2KT, EIL).

#### 3.1.1 Text generation quality

As detailed in Table 1, ACE-Net demonstrates a robust lead across standard NLG metrics. Notably, compared to the traditional soft-attention baseline (SAT-ResNet101), our model achieves a substantial leap in BLEU-4 (0.7511). This performance gap suggests that while single-layer attention mechanisms often struggle to capture the syntax of fine-grained pathology, the proposed Triple-Guided MoE strategy successfully models more complex linguistic structures. Perhaps most telling is the dramatic improvement in the METEOR metric (reaching 0.8611). We attribute this directly to the evidential gating mechanism: by filtering visual noise at the encoding stage, the model likely achieves better semantic coverage, ensuring that generated words map more accurately to visual facts rather than artifacts. Furthermore, the 7% gain in ROUGE-L over our prior work, EIL, validates the hypothesis that co-training can effectively harness unlabeled data to improve generalization.

**Table 1:** Performance comparison of different methods on the test set for text generation metrics. The best results are highlighted in **bold**.

Methods	Backbone	BLEU-4	ROUGE-L	METEOR
SAT (VGG-19)	VGG-19	0.4697	0.5921	0.7916
SAT (ResNet-101)	ResNet-101	0.5064	0.6047	0.7786
R2Gen	ResNet-101	0.3714	0.5982	0.3045
R2GenCNN	ResNet-101	0.4352	0.6125	0.3675
M2KT	ResNet-101	0.6340	0.8208	0.8151
EIL	ResNet-101	0.7346	0.8040	0.5013
**ACE-Net (Ours)**	**ResNet-101**	**0.7511**	**0.8728**	**0.8611**

#### 3.1.2 Clinical accuracy and safety

Beyond linguistic fluency, the “safety profile” of a medical AI is defined by its ability to correctly identify pathology. Table 2 highlights a critical trade-off—or rather, the resolution of one. While ACE-Net maintains high Precision (0.848), it achieves a notable improvement in Recall (0.818) compared to EIL. Clinically, this balance is paramount: elevated recall implies a reduced risk of missing subtle lesions (such as early perforations), while stable precision confirms that our uncertainty module effectively suppresses the “hallucinations” that often plague generative models.

**Table 2:** Comparison of clinical pathological keyword classification performance.

Methods	Precision	Recall	F1-Score
EIL	0.839	0.625	0.717
**ACE-Net (Ours)**	**0.848**	**0.818**	**0.832**

Bold values indicate the best performance among the compared methods for each metric.

#### 3.1.3 Cross-domain generalization and data efficiency on IU-Xray

To comprehensively evaluate the cross-domain generalizability of ACE-Net, we conducted comparative experiments on the public IU-Xray dataset. As shown in Table 3, we compared our model with several state-of-the-art (SOTA) methods, including R2Gen, AlignTransformer, EIL, METransformer, and KIUT.

**Table 3:** Quantitative comparison with state-of-the-art methods on the public IU-Xray dataset. To evaluate data efficiency, ACE-Net is co-trained utilizing only 35% labeled training data, whereas most other baselines utilize 100% fully supervised data.

Methods	Data utilization	BLEU-4	ROUGE-L	METEOR
R2Gen	100% labeled train set	0.165	0.371	0.187
AlignTransformer	100% labeled train set	0.173	0.379	0.204
EIL	Human-in-the-loop	0.157	0.356	0.162
METransformer	100% labeled train set	0.172	0.380	0.192
KIUT	100% labeled train set	**0.185**	**0.409**	0.242
**ACE-Net (Ours)**	**35% Labeled train data**	0.104	0.385	**0.331**

Bold values indicate the best performance for each metric.

**Table 4:** Ablation study of ACE-Net. We analyze the contribution of each component by selectively removing them. Checkmarks (✓) indicate the inclusion of a specific module. “Co-T” denotes Co-training; “$L_{attn}$” denotes Attention Consistency Loss.

Model	Components						Metrics			
	Co-T	AAM	MoE	Spatial	Uncert.	$L_{attn}$	BLEU-4	ROUGE-L	METEOR	F1
Model A	✓	✓	✓	✓		0.6973	0.8460	0.8281	0.8197	
Model B	✓		✓	✓	✓		0.7458	0.8632	0.8501	0.8289
Model F	✓	✓		✓	✓	✓	0.6981	0.8437	0.8221	0.8223
Model G	✓	✓	✓		✓	✓	0.7267	0.8602	0.8444	0.8212
Model E	✓	✓	✓	✓		✓	0.7218	0.8575	0.8386	0.8277
Model D	✓	✓	✓	✓	✓		0.7139	0.8476	0.8314	0.8289
**Model C (Ours)**	**✓**	**✓**	**✓**	**✓**	**✓**	**✓**	**0.7511**	**0.8728**	**0.8611**	**0.8324**

Bold values indicate the best performance among the ablation settings for each metric.

Fully supervised SOTA models demonstrate strong performance in strict N-gram matching metrics (such as BLEU), as they can leverage 100% of the labeled training data to learn the complete distribution of standard report expressions. In this work, however, we aim to explore the model's potential in data-scarce clinical scenarios. Under a strict semi-supervised setting, ACE-Net utilizes only 35% of the total dataset for text-level supervision (derived from 70% of the 50% labeled subset).

Despite this limited supervision, ACE-Net achieves a highly competitive ROUGE-L score (0.385) and makes a significant breakthrough in the semantic-focused METEOR metric (0.331). This advantage indicates that, facilitated by evidential gating and spatial consistency constraints, our framework can effectively capture essential clinical semantics and anatomical correlations without relying on massive annotated data, thus offering a practical and data-efficient solution for specialized medical domains.

### 3.2 Ablation study: mechanism deconstruction

To dissect the contribution of individual components, we conducted a step-wise ablation study (Table 3).

**1. The power of co-training**. Comparing Model A and Model B, the transition from supervised-only learning to semi-supervised co-training yields a clear performance lift (+6.9% in BLEU-4). This confirms that regularizing the feature space with unstructured data helps mitigate the data scarcity bottleneck inherent in medical imaging.

**2. The “spatial noise” phenomenon**. A counter-intuitive finding emerged when comparing Models B, D, and C. We observed that adding anatomical attention without consistency constraints (Model D) actually degraded performance compared to having no attention at all (Model B). This suggests that in a weak supervision setting, unconstrained attention maps can degenerate into “spatial noise,” distracting the decoder. Model C (ACE-Net) reverses this by introducing the Spatial Consistency Alignment ($L_{SSL}$), which acts as a regularizer to force these disordered maps into meaningful localization signals.

**3. Gating and experts**. Removing the uncertainty mechanism (Model E) resulted in a measurable drop, reaffirming the role of evidential soft gating as a necessary “confidence filter.” Similarly, the advantage of Model C over Model F supports the use of Mixture-of-Experts (MoE) to handle the heterogeneous nature of diagnostic descriptions.

### 3.3 Qualitative analysis and discussion

#### 3.3.1 Interpreting high metric scores

It is prudent to contextualize the absolute values of our reported metrics. As seen in the results, most methods achieve relatively high scores (e.g., BLEU-4 > 0.70). This is partly attributable to the domain specificity of otolaryngology reports, which tend to follow standardized, concise syntactic templates (e.g., “Tympanic membrane is intact”) rather than the complex narratives found in chest radiography. However, ACE-Net's superior F1-Score indicates that its performance is driven by fine-grained semantic distinctions, not merely by memorizing these templates.

#### 3.3.2 Visualization of implicit anatomical localization

**Figure 4:**
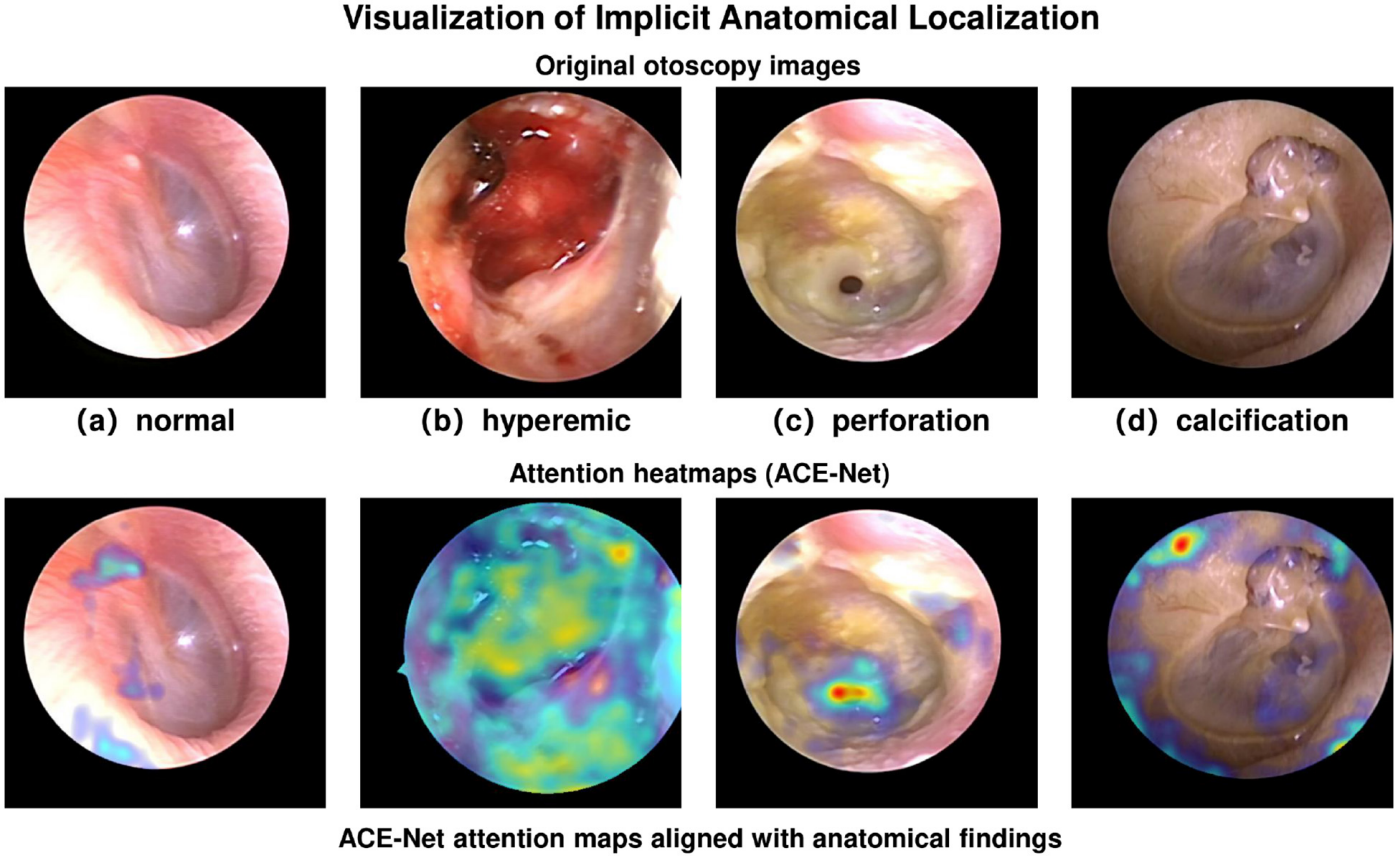
Visualization of implicit anatomical localization results. The top row displays original otoscopy images presenting various conditions, including normal tympanic membranes, perforations, and inflammation. The bottom row shows the corresponding attention heatmaps generated by ACE-Net. The results indicate that the model's focus regions maintain spatial consistency with pathological targets, achieved without pixel-level supervision.

To verify that the model is truly “looking” at the pathology, we visualized the attention heatmaps in Figure 4.

**Figure 5:**
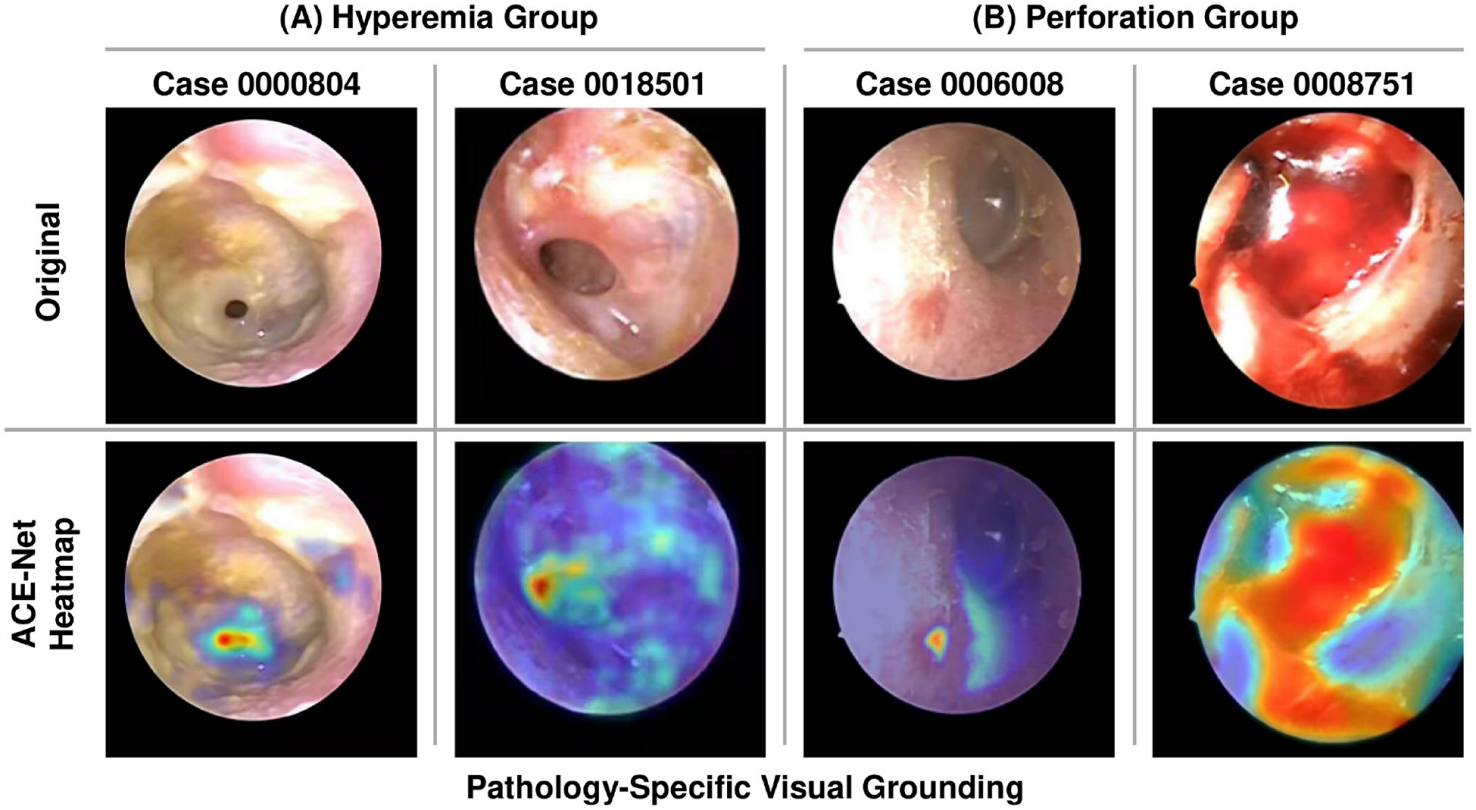
Qualitative evidence of anatomical grounding on hallmark pathologies. The first two columns (e.g., Case 0000804) demonstrate ACE-Net's capability to capture diffuse inflammatory patterns such as **hyperemia** and blood accumulation in the external canal. The last two columns showcase the precise localization of structural defects, particularly the **small perforation** in the anteroinferior quadrant (e.g., Case 0006008), confirming the model's reliability in grounding diagnostic keywords to authentic clinical targets.

To further investigate the clinical interpretability of ACE-Net and its resistance to “diagnostic hallucinations,” we visualized the attention mechanisms on two hallmark conditions: *hyperemia* and *perforation* (Figure 5).

In cases of diffuse hyperemia (Columns 1–2), the Anatomical Attention Maps (AAMs) effectively encapsulate the widespread vascular congestion and blood accumulation in the canal. More significantly, for structural breaches (Columns 3–4), the network accurately centers its focus on the *anteroinferior quadrant* where the small perforation is located (e.g., Case 0006008). These results confirm that ACE-Net effectively induces anatomical grounding, ensuring that the generated diagnostic keywords are supported by precise visual evidence rather than spurious correlations.

## 4 Discussion

This research aims to address the contradiction between data scarcity and the urgent clinical need for accurate and reliable diagnostic reports. In ACE-Net, we introduce a semi-supervised framework capable of effectively mining reliable anatomical features from unstructured data, thus eliminating the traditional reliance on expensive pixel-level annotations. Furthermore, by combining an evidence-driven soft-gating mechanism with a triple-guided expert hybrid decoder, we demonstrate that high-quality diagnostic reports can be generated through global supervision under appropriate uncertainty constraints. Our empirical analysis reveals a key finding: attention mechanisms are inherently unstable in weakly supervised environments. Specifically, we observe that in the absence of explicit constraints, these mechanisms readily degenerate into “spatial noise,” failing to capture meaningful anatomical structures. Our proposed spatial consistency alignment strategy effectively addresses this issue, acting as a crucial regularization mechanism to transform disordered attention patterns into meaningful, implicit localization signals.

From a clinical perspective, this advances the frontiers of trustworthy artificial intelligence. ACE-Net's ability to estimate uncertainty enables conservative inferences, proactively suppressing unrealistic descriptions based on vague visual evidence, thereby improving the safety of computer-aided diagnostic systems.

Looking ahead, we have identified two promising directions for development. First, while our framework performs well with two-dimensional endoscopic images, extending the theory of uncertainty of evidence to **three-dimensional volumetric data** (such as CT or MRI sequences) provides a pathway to handling more complex spatial dependencies. Second, although the MoE architecture successfully captures a wide range of pathological manifestations, its “knowledge horizon” remains limited by the training dataset. Future work will explore integrating the encyclopedic medical knowledge of **large visual language models** into expert modules to enhance generalization capabilities for rare and long-tailed diseases.

## Data Availability

The clinical endoscopy dataset utilized in this study was provided by Ya'an People's Hospital. Due to strict privacy regulations governing sensitive medical data and its continued use in ongoing institutional research, the dataset is not publicly available at this time. Inquiries regarding data access for verification purposes may be directed to the corresponding authors, subject to ethical approval and data-sharing agreements.
